# Supplementary material for: Gene Ontology Meta Annotator for Plants (GOMAP)
Source: Plant Methods. 2021 May 25;17:54. doi: 10.1186/s13007-021-00754-1 (PMC8146647; doi:10.1186/s13007-021-00754-1)
Supplement: Supplementary file 1 — Additional file 1. Additional figures and tables. [file 13007_2021_754_MOESM1_ESM.pdf]

GOMAP Supplemental Data

Kokulapalan Wimalanathan

2/23/2021

Supplemental Tables

Table ST1: Location of input fasta files for the maize lines

Inbred Line	Link
B73v4	ftp://ftp.ensemblgenomes.org/pub/plants/release-32/fasta/zea_mays/pep/Zea_mays.AGPv4.pep.all.fa.gz
B73v3	https://ftp.maizegdb.orgGDB/FTP/B73_RefGen_v3/Zea_mays.AGPv3.22.pep.all.fa.gz
Mo17	https://ftp.maizegdb.orgGDB/FTP/Zm-Mo17-REFERENCE-CAU-1.0/Zm00014a.proteins.fa.gz
PH207	https://ftp.maizegdb.orgGDB/FTP/Zm-PH207-REFERENCE_NS-UIUC_UMN-1.0/Zm00008a.protein.fa.gz
W22	https://ftp.maizegdb.orgGDB/FTP/Zm-W22-REFERENCE-NRGENE-2.0/Zm00004b.protein.fa.gz

Table ST2: Comparison of GOMAP Annotations and Plant-Specific Subset

		Cellular Component		Molecular Function		Biologial Process	
inbred	source	All	Plant-specific	All	Plant-specific	All	Plant-specific
B73v3	GOMAP	135,251	133,272	87,953	87,267	291,855	287,199
B73v4	Community	35,771	35,764	49,659	49,685	44,998	45,016
B73v4	GOMAP	88,831	87,683	82,849	81,906	278,952	270,146
Mo17	GOMAP	87,573	86,482	79,755	78,892	278,043	269,274
PH207	Community	4,429	4,428	23,192	23,219	13,093	13,081
PH207	GOMAP	90,625	89,511	86,106	85,151	288,937	278,278
W22	GOMAP	95,397	94,271	85,616	84,699	290,032	280,705

Figure S1: Distribution of Specificity for the maize annotations from community and GOMAP

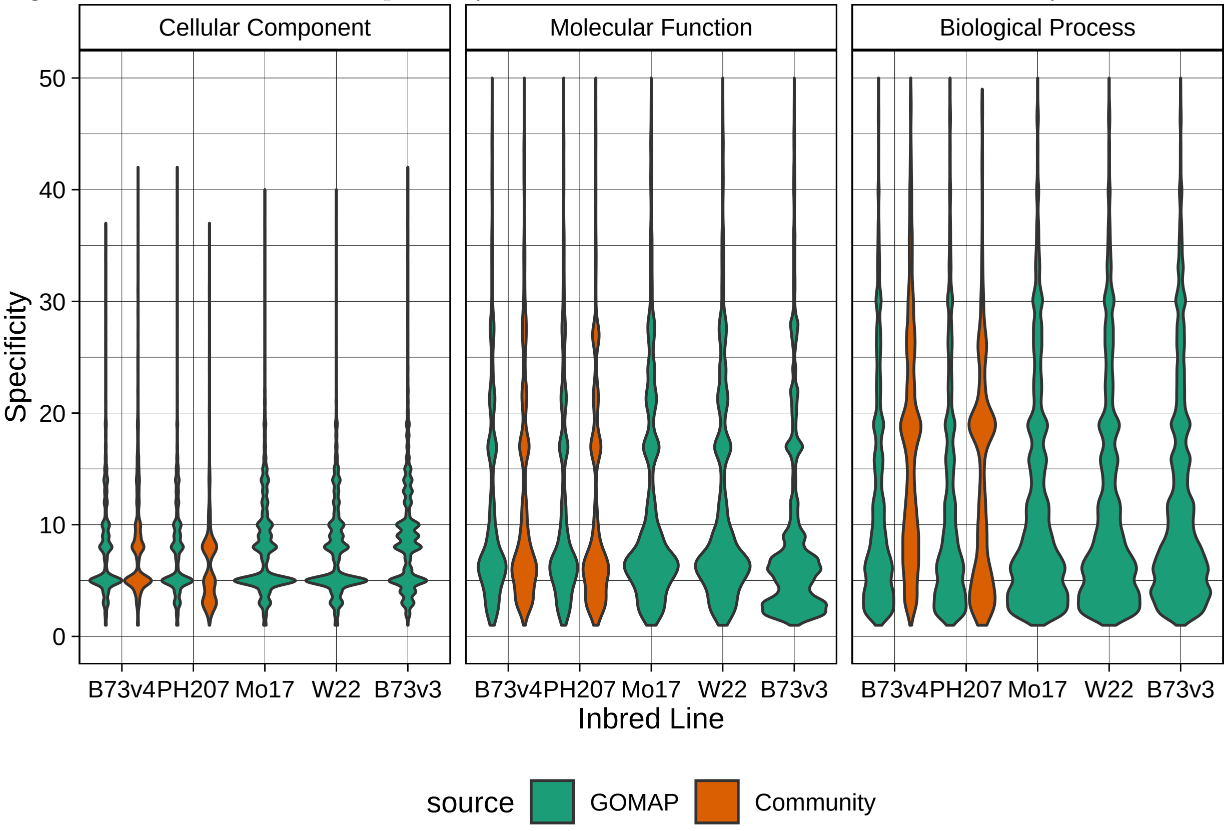

Left column: Cellular Component. Middle column: Molecular Function. Right column: Biological Process. Inbred lines are denoted along the x-axis. The specificity is denoted along y-axis, and the range is limited to less than 50. GOMAP annotations are denoted by green color. Community annotations are denoted by a orange color. The width of the plot along the y-axis indicates the proportion of the annotations that particular specificity value.

Figure S2: Distribution of Specificity for the genes that are shared between community and GOMAP

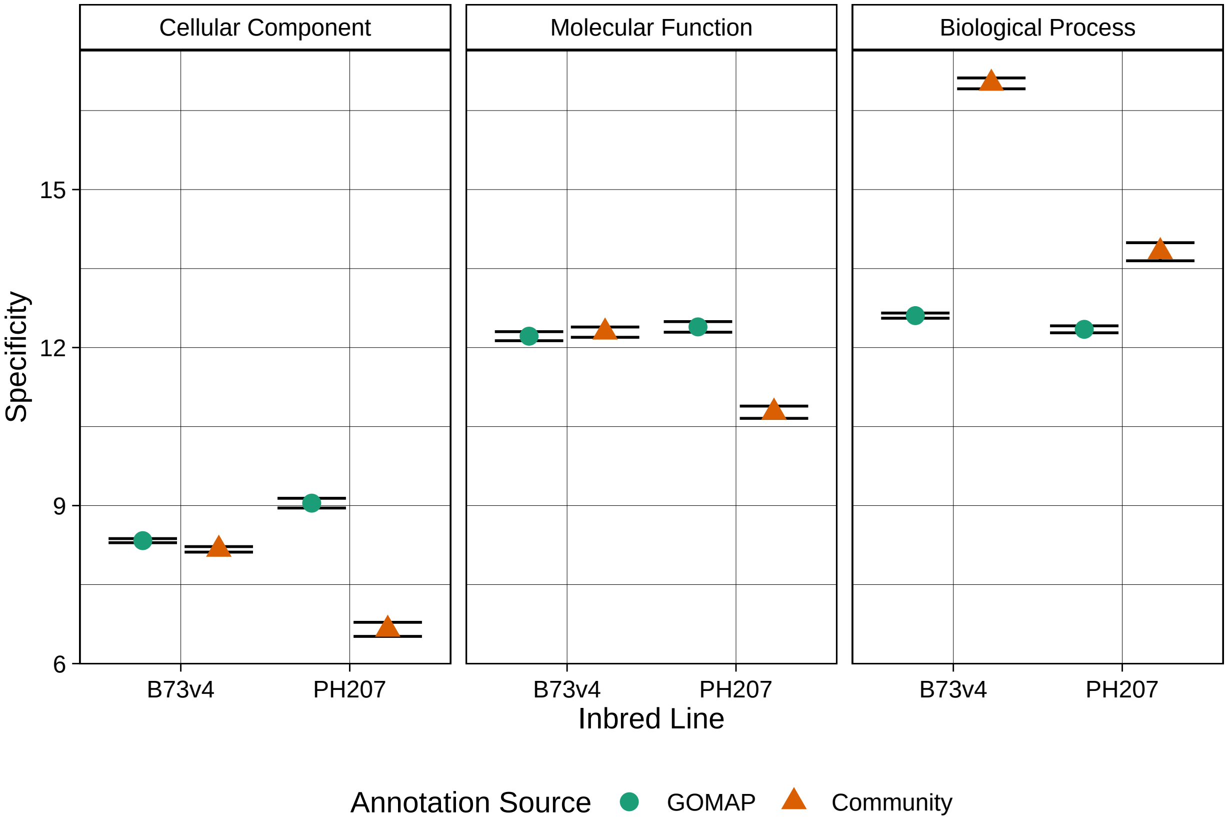

Left column: Cellular Component. Middle column: Molecular Function. Right column: Biological Process. Inbred lines are denoted along the x-axis. The specificity is denoted along y-axis, and the range is limited to less than 50. GOMAP annotations are denoted by green color. Community annotations are denoted by a orange color. The width of the plot along the y-axis indicates the proportion of the annotations that particular specificity value.
